# Suicide Precipitants Differ Across the Lifespan but Are Not Significant in Predicting Medically Severe Attempts

**DOI:** 10.3390/ijerph15040691

**Published:** 2018-04-05

**Authors:** Carol C. Choo, Peter K. H. Chew, Roger C. Ho

**Affiliations:** 1 College of Healthcare Sciences, James Cook University, Singapore 387380, Singapore; peter.chew@jcu.edu.au; 2 Department of Psychological Medicine, Yong Loo Lin School of Medicine, National University of Singapore, Singapore 119007, Singapore; pcmrhcm@nus.edu.sg

**Keywords:** suicide precipitants, lifespan, medical severity

## Abstract

An important risk factor for suicide assessment is the suicide precipitant. This study explores suicide attempt precipitants across the lifespan. Three years of medical records related to suicide attempters who were admitted to the emergency department of a large teaching hospital in Singapore were subjected to analysis. These cases were divided into three age groups: Adolescence, Early Adulthood, and Middle Adulthood. A total of 540 cases were examined (70.9% females; 63.7% Chinese, 13.7% Malays, 15.9% Indians), whose ages ranged from 12 to 62. There were eight cases above the age of 65 years which were excluded from the analysis. Significant differences were found in precipitants for suicide attempts across the lifespan. Middle adults had relatively fewer relationship problems, and adolescents had comparatively fewer financial and medical problems. The models to predict medically severe attempts across the age groups using suicide precipitants were not significant. The findings were discussed in regards to implications in suicide assessment and primary prevention in Singapore, as well as limitations and recommendations for future research.

## 1. Introduction

Worldwide trends indicated that suicide rates increased with advancing age from adolescence to adulthood [1,2,3]. This trend was often attributed to the usage of more lethal methods by older suicide attempters [4]. Further analysis of age trends revealed considerable cross-national variability and interesting differences between Western and Asian countries [5]. One example is that suicide rates in Australia and the United States were typified by upward-sloping graphs, whereas in Japan, the pattern was bimodal, and recently became trimodal, with a middle-age peak. The bimodal trend was evident in early studies done in Singapore [6], but recently gradually dissipated [7]. In Asian countries, e.g., Hong Kong and Singapore, adolescent suicide rates were lower than adult rates in the recent years, which might be attributed to improvements in economic conditions [8]. However, a recent Singaporean study found a marked increase in suicide attempts in Asian youths as compared to other age groups [9]. These findings highlighted the importance of a more in-depth analysis of age trends in suicides to facilitate better understanding of such trends.

Age plays an important role in determining suicide precipitants, which are the problems encountered shortly before suicide [10]. In Asia, precipitants for youth suicides included interpersonal conflicts, stress over military service [11,12,13,14], family problems [11,15,16], and academic stress [17]. Use of substance and alcohol could also increase existing suicide risk in vulnerable youths [18,19], although this phenomenon was less prevalent for Asian youths [9], as compared with their Western counterparts [18]. By middle age, the societal and cultural expectations in Asian societies were that individuals had established themselves in their careers, with a stable family and financial security, those who had achieved little in these milestones might face disappointment and frustration [10]. Major reasons for suicides included financial and marital problems in middle adulthood, and physical illnesses in the elderly. Elderly suicides had been more prevalent in the lower socioeconomic strata [20], and associated with physical illness [10], financial problems, and social isolation.

Suicide attempters often react impulsively to a stressor [21], and their suicide notes are charged with negative emotions [13]. When examined closely, a myriad of factors precipitate people to suicide across the age groups. However, explanations of the mechanisms precipitating suicide across the lifespan remain inadequate, as many biopsychosocial factors interact and lead to suicide attempts [22,23], so suicide epidemiology needs to include an expanded interdisciplinary perspective, with due consideration of biopsychosocial patterns of transition from adolescence into adulthood, and changes in traditional Asian family configurations, which have greatly altered in recent times. One example is the recognition that psychosocial risk factors for adolescent suicide attempts have changed in recent times, to include stressors stemming from homosexual orientation, and the developmental timing of puberty [24], as sexual development is commencing earlier but socioeconomic independence is being reached later, so adolescents and young adults may experience more stress from relationship issues, as compared with earlier decades. Traditional Asian family configurations had also changed in the recent decades, with more adult children choosing not to live with their parents, leaving older people on their own, which might add to existing suicide risk [10]. The breakdown of traditional extended family systems and inadequate social support were postulated to account for elderly suicides in Asia [13,25].

Most suicide studies were conducted on suicide deaths, the current study on suicide attempts is unique in that it seeks to enhance our understanding of precipitants contributing to medically severe attempts across the lifespan. Analysis of suicide precipitants and prediction of medically severe attempts across the lifespan could help further our understanding with the ultimate aim to prevent medically severe attempts resulting in death, and to inform more targeted strategies to resolve the precipitants across the lifespan.

This study aimed to analyze the differences in suicide precipitants across the lifespan, and the prediction of medically severe attempts over the lifespan using available suicide precipitants. It was hypothesized that suicide precipitants and medical severity of suicide attempts would differ across the lifespan. Based on evidence in both Western and Asian studies, analysis would be conducted on the following variables, available as part of the Suicide Risk Assessment Form utilized by medical officers at the local hospital where this study took place. The precipitants included: medical problems [26], work/academic problems [17], family problems [27], relationship problems [28], and financial problems [29].

## 2. Materials and Methods

### Procedure

Ethics approval was obtained from the Domains-Specific Review Board of a large teaching hospital in Singapore (2009/00595) and the Human Research Ethics Committee at James Cook University (H3445). This study was based on an archival retrospective review of de-identified hospital records of patients who were admitted for a suicide attempt from January 2004 to December 2006. Data were collected from multiple hospital databases related to the suicide attempters who were admitted over the three-year period and this data set was the most comprehensive data set available from the hospital, as such assessment data were not collected prior to and following the stipulated period. Archival data were extracted from the Patient Psychiatric Assessment Form (PPAF). The PPAF included the Suicide Risk Assessment form, information about the current suicide attempt, as well as information about the patient, and suicide precipitants.

All cases of attempted suicide were assessed by medical officers in the emergency department under the supervision of a consultant psychiatrist, and the interview took approximately 20 min. This assessment was part of the protocol standard operating procedure for patients admitted following a medically treated suicide attempt.

The inclusion criterion for the current study were patients who were admitted to the emergency department from January 2004 to December 2006 and were assessed by medical officers using the PPAF. There were a total of 671 cases examined in the study. Cases with missing data on key variables were removed from the data set (*n* = 123), resulting in a sample of 548 cases. These cases were divided into the following developmental stages: Adolescence (12 to 18 years), Early Adulthood (19 to 34 years), Middle Adulthood (35 to 64 years), and Late Adulthood (65 years and above) [30]. As there were only eight participants in the Late Adulthood group, these cases were removed from the data set, resulting in a final sample of 540 cases (70.9% females; 63.7% Chinese, 13.7% Malays, 15.9% Indians, and 6.7% others). Adolescents (*n* = 109) have an age range of 12 to 18 (M = 15.95, SD = 1.51), Early Adults (*n* = 260) have an age range of 19 to 34 (M = 25.57, SD = 4.72), and the Middle Adults (*n* = 171) have an age range of 35 to 62 (M = 43.60, SD = 6.82).

## 3. Measures

### Suicide Risk Assessment Form

The Suicide Risk Assessment Form (SRAF) is a 2-page questionnaire designed to be conducted as a semi-structured interview by medical officers. The content of the assessment form included: demographic information, the presence of stressors (e.g., work, family, relationships, financial, medical) on dichotomous scales, and medical officer’s clinical judgment of the medical severity of the attempt on a 4-point scale (high, moderate, low, no actual attempt). “High” and “moderate” were recoded into “severe” and “low” and “no attempt” were recoded into “not severe”. This checklist was devised for the collation of information deemed important for clinical usage in suicide risk assessment and management planning, and psychometric properties were not available.

## 4. Data Analysis and Results

### 4.1. Data Analysis

The data were analyzed using IBM SPSS Statistics 23 (IBM, Armonk, NY, USA). A series of six Chi-square test of independence was conducted to examine the association between age groups (Adolescence, Early Adulthood, and Middle Adulthood) with suicide precipitants (i.e., work, family, relationship, financial, and medical problems) and the medical severity of suicide attempts. Due to the number of analyses conducted, the alpha level was controlled, using Bonferroni adjustment (i.e., adjusted alpha = 0.05/6 analyses = 0.008). Subsequently, three direct logistic regressions were performed, one for each age group (Adolescence, Early Adulthood, and Middle Adulthood), to assess the effects of work, family, relationship, financial, and medical problems on the likelihood that participants attempted medically severe suicide attempts.

### 4.2. Results

The percentages of suicide cases with work, family, relationship, financial, and medical problems, and medically severe suicide attempts are presented in Table 1. A series of six Chi-square test of independence was conducted to examine the association between the age groups and those variables. The results are presented in Table 1. There was a significant association between the age groups and relationships problems, with the middle adults having less relationship problems than the other two groups. There was a significant association between the groups and financial problems, with the adolescents having less financial problems than the other two groups. Lastly, there was a significant association between the groups and medical problems, with the adolescents having less medical problems then the other two groups.

Three direct logistic regressions were performed, one for each group, to assess the effects of work, family, relationship, financial, and medical problems on the likelihood that participants would attempt medically severe suicide. The model contained five independent variables (see Table 1). The full model containing all predictors was not statistically significant for adolescents, χ^2^ (5, *N* = 109) = 3.97, *p* > 0.05, early adults, χ^2^ (5, *N* = 260) = 7.40, *p* > 0.05, and middle adults, χ^2^ (5, *N* = 171) = 8.90, *p* > 0.05, indicating that the model was unable to distinguish between those who attempted medically severe suicide and those who attempted medically non-severe suicide.

## 5. Discussion

This study aimed to explore differences in suicide precipitants for suicide attempts across the lifespan in Singapore, and prediction of medically severe suicide attempts across the lifespan. Three years of medical records of 540 suicide attempters were analyzed. As hypothesized, differences in suicide precipitants were seen across the lifespan from adolescence to adulthood. There was a significant association between the age groups and relationship problems, with the middle adults having less relationship problems than the other two groups. There was also a significant association between the groups with medical and financial problems, with the adolescents having less financial and medical problems than the other two groups. The trend in financial and medical problems is consistent with previous research reporting that financial problems and chronic disease were more common in older suicide attempters [29]. The trend for relationship problem is consistent with previous research reporting the association between interpersonal stress and suicidal ideation in adolescence [28], and early adults might lack the capacity to overcome interpersonal conflicts, and tended to become depressed, angry and alienated in the face of these relationship stressors [21]. By middle adulthood, they might have established stable families [10], or learnt ways to cope with relationship stressors [31]. An understanding of precipitants across the lifespan could deepen clinicians’ insight of such dynamic suicide risk factors, and inform targeted interventions to reduce further attempts, which could include interventions informed by Dialectical Behavioural Therapy, or strategies to enhance coping skills [31].

In the current study, the middle adulthood group spanned from 35 to 64 years. There were limitations in the current study in that the cases in this age group could be affected by a myriad of factors, so further analysis could be conducted for this age group, e.g., some cases in the group might have retired but some of them would still be employed, some of them might be married, and living with their families, however, such data were not gathered. Future research could collect detailed information to aid in further analysis of the factors affecting adults in this age group. In Asian countries, there is limited government support, particularly for those with sickness and physical handicap [10], and the support for sick parents normally falls to their adult children. Recent trends for adult children to not live with their parents leave older people on their own which might contribute to suicide risk [10,21]. However, availability of welfare supports and government subsidies might help ameliorate the problems [32].

The finding of non-significant models predicting medically severe suicide attempts using suicide precipitants for each of the age groups is not surprising. Deeper analysis of local literature is helpful in our understanding of the non-significant results. There might be latent variables, such as suicide intent [33], and interaction effects between age, gender, ethnicity [9,34] and suicide precipitants that were not captured in the current study. A large scale local study found that more males than females in the 25 to 44 years age group committed suicide due to financial problems, but for the youths below 24 years old and the elderly above 60 years old, more females than males committed suicide due to financial problems [21]. More elderly male suicides suffered from physical illness, but more female suicides were associated with interpersonal problems across all ages. Suicide precipitants in young males included incapacitating mental and physical illness, academic stress and unemployment [15]. In Singapore, every male citizen and permanent resident above 18 years old had to do compulsory military service, and adjustment to military life was cited as a major stressor [12]. In contrast, marital problems were significant in precipitating suicide in young Asian females [15,21]. In addition, there could be interaction effects between suicide precipitants, ethnicity [34], age [9], with a range of suicide risk and protective factors to contribute to medically severe attempts. Protective factors might contribute to resilience and help to buffer again stressors [9,34]. However, most of these protective variables were not included in the current study, and could be included in future research, with a longitudinal design. Previous research indicated that despite strong cross-sectional associations, resilience and suicidal ideation appeared to be unrelated in a longitudinal context [35], and could be investigated further. A recent local study found that Chinese made up the largest proportion while Indians made up the lowest proportion of medically severe attempts, although this difference was not significant [34], possible interactions between age, ethnicity and gender could be further explored in their contribution to medical severity.

The sections above highlighted many limitations in the current study in that data concerning marital, socioeconomic and employment status, eligibility for government subsidies, living conditions, specific information regarding the nature of the stressors, whether stresses were chronic or episodic, and the interactions between the various biopsychosocial factors were not fully captured, but could be considered in future research. Further statistical analysis as well as in-depth qualitative interviews might uncover intricate interaction patterns between age, gender and ethnicity, suicide precipitants and other risk and protective factors in medically severe suicide attempts. Possible mediating factors might include presence of dependents, coping strategies, religious beliefs, and other factors that might be protective during times of heightened stress [34]. In a recent study [34], high perceived lethality in suicide attempts for Chinese attempters was found to be predicted by suicide intent and low opportunity for rescue. It is possible that regardless of suicide precipitants, the presence of a potential rescuer could help to reduce the lethality of the attempt.

The findings that there were differences in suicide precipitants across the age groups have implications for suicide interventions and primary prevention. Interventions could be more targeted. Suicide prevention efforts could be targeted at problem solving strategies to resolve the precipitants identified across the age groups. Social work agencies and financial services could work with general practitioners and nursing staff at local polyclinics and medical clinics to help early and middle adult patients with financial problems [30], and consider using a screening tool e.g., PHQ-9 to routinely screen for mental health problems and suicidal ideation [36] in patients with medical problems. Counselling and crisis interventions could be more targeted towards resolution of precipitants, e.g., coping with relationship stressors in adolescents and early adults. Such strategies focusing on problem solving skills are consistent with interventions suggested by previous literature for suicide prevention in Asians [9,34,37].

Additional limitations to the study included the following: a lack of a control group limited how the data could be interpreted. Future research could compare data between suicide attempters and matched controls in the normal population for better interpretation of data. Other limitations of the study included the reliance on self-report and the brief nature of the assessment, as well as the usage of single items on dichotomous scales, which placed constraints on the depth of the information obtained. With the consent of the patients, collateral information from family members could be collected, and in-depth interviews with patients could be analyzed with a qualitative paradigm. In the study, most of the cases were in the relatively younger age groups, and there were 8 cases above the age of 65 years which were excluded from the analysis. However, elderly suicides are a rising concern and current understanding is lacking [38]. Future research could consider in-depth interviews with elderly suicide attempters to facilitate a full understanding.

## 6. Conclusions

In conclusion, the findings have implications for informing our efforts on suicide assessment and primary prevention across the lifespan. By using age-specific assessments and interventions that are substantiated by empirical findings from current research in the local population, the clinician will be taking a step forward in utilizing the scientist-practitioner model in their evidence based practice. The current study has highlighted contribution from different categories of suicide precipitants across the lifespan. Interpreted together with a review of relevant local literature in the area, the current study suggests that suicide assessment and interventions could take into account greater consideration of a range of biopsychosocial factors e.g., ethnicity, gender, and risk and protective factors rather than suicide precipitants alone. This study adds to the current literature on suicide research, and draws further focus to the importance of training competent clinicians who are able to conduct full and comprehensive biopsychosocial suicide risk assessment, as well as the importance of screening at-risk patients across the lifespan.

## Figures and Tables

**Table 1:** Percentage of Participants with Work, Family, Relationship, Financial, and Medical Problems, and Medically Severe Suicide Attempts.

Variables	Adolescence		Early Adulthood		Middle Adulthood		*χ*^2^	Cramer’s V
	Yes	No	Yes	No	Yes	No		
**Predictors**								
(1) Work/Academic	29.4	70.6	41.5	58.5	38.6	61.4	4.85	-
(2) Family	57.8	42.2	41.9	58.1	44.4	55.6	8.02	-
(3) Relationship	63.3	36.7	63.8	36.2	48.5	51.5	11.08 *	0.14
(4) Financial	9.2	90.8	22.7	77.3	37.4	62.6	29.64 *	0.23
(5) Medical	6.4	93.6	12.3	87.7	27.5	72.5	26.96 *	0.22
**Criterion**								
(1) Medically Severe(Severe/Not Severe)	54.1	45.9	19.6	50.4	49.7	50.3	0.70	-

*df* = 2. Due to the number of analyses conducted, the alpha level was controlled for using the Bonferroni adjustment (i.e., adjusted alpha = 0.05/6 analyses = 0.008). * *p* < 0.008.
